# Construction and characterization of recombinant flaviviruses bearing insertions between E and NS1 genes

**DOI:** 10.1186/1743-422X-4-115

**Published:** 2007-10-30

**Authors:** Myrna C Bonaldo, Samanta M Mello, Gisela F Trindade, Aymara A Rangel, Adriana S Duarte, Prisciliana J Oliveira, Marcos S Freire, Claire F Kubelka, Ricardo Galler

**Affiliations:** 1 Fundação Oswaldo Cruz, Instituto Oswaldo Cruz, Laboratório de Biologia Molecular, de Flavivírus, Rio de Janeiro, Fundação Oswaldo Cruz. Avenida Brasil 4365, Manguinhos, Rio de Janeiro, RJ 21045-900, Brazil; 2 Fundação Oswaldo Cruz, Instituto de Tecnologia em Imunobiológicos, Rio de Janeiro, Brazil; 3 Fundação Oswaldo Cruz, Instituto Oswaldo Cruz, Laboratório de Imunologia Viral, Rio de Janeiro, Brazil

## Abstract

**Background:**

The yellow fever virus, a member of the genus *Flavivirus*, is an arthropod-borne pathogen causing severe disease in humans. The attenuated yellow fever 17D virus strain has been used for human vaccination for 70 years and has several characteristics that are desirable for the development of new, live attenuated vaccines. We described here a methodology to construct a viable, and immunogenic recombinant yellow fever 17D virus expressing a green fluorescent protein variant (EGFP). This approach took into account the presence of functional motifs and amino acid sequence conservation flanking the E and NS1 intergenic region to duplicate and fuse them to the exogenous gene and thereby allow the correct processing of the viral polyprotein precursor.

**Results:**

YF 17D EGFP recombinant virus was grew in Vero cells and reached a peak titer of approximately 6.45 ± 0.4 log10 PFU/mL at 96 hours post-infection. Immunoprecipitation and confocal laser scanning microscopy demonstrated the expression of the EGFP, which was retained in the endoplasmic reticulum and not secreted from infected cells. The association with the ER compartment did not interfere with YF assembly, since the recombinant virus was fully competent to replicate and exit the cell. This virus was genetically stable up to the tenth serial passage in Vero cells. The recombinant virus was capable to elicit a neutralizing antibody response to YF and antibodies to EGFP as evidenced by an ELISA test. The applicability of this cloning strategy to clone gene foreign sequences in other flavivirus genomes was demonstrated by the construction of a chimeric recombinant YF 17D/DEN4 virus.

**Conclusion:**

This system is likely to be useful for a broader live attenuated YF 17D virus-based vaccine development for human diseases. Moreover, insertion of foreign genes into the flavivirus genome may also allow *in vivo *studies on flavivirus cell and tissue tropism as well as cellular processes related to flavivirus infection.

## Background

The yellow fever 17D virus is attenuated and used for human vaccination for 70 years. Some of the outstanding properties of this vaccine include limited viral replication in the host but with significant expansion and dissemination of the viral mass yielding a robust and long-lived immune response [1]. It also induces a significant T cell response [2-5]. The vaccine is cheap, applied in a single dose and involves well-established production methodology and quality control procedures, which include monkey neurovirulence assay. Altogether, the YF 17D virus has become very attractive as an expression vector for the development of new live attenuated vaccines [6,7].

The development of infectious clone technology has allowed the genetic manipulation of the YF 17D genome, towards the expression of foreign genes. Different technical approaches to constructing recombinant viruses based on the YF 17D virus are [6,8] possible and will vary according to the antigen to be expressed. One major approach has been the creation of chimeric viruses through the exchange of structural prM/M/E genes [9]. Another advance has been the expression of particular foreign epitopes in the *fg *loop of the E protein [6,8]. Heterologous epitopes have also been inserted between the nonstructural proteins by flanking them with proteolytic cleavage sites specific for the viral NS2B-NS3 protease [10]. Such a strategy was tested for all sites cleaved by the viral protease, but only three of these positions, the amino-terminus, and the C-prM and NS2B-NS3 intergenic regions yielded viable viruses. Recombinant YF 17D viruses with insertions between NS2B-3 replicated best [10] and this methodology has been further exploited [4,11].

Based on the natural length variation, the 3' untranslated region of flaviviruses [12] has been subjected to the insertion of genetic cassetes containing internal ribosomal entry sites (IRES) from picornaviruses and reporter genes [13]. However, genetic instability in this region resulted in partial elimination of the cassete [1,14].

The development of flavivirus replicon technology allowed for the transient expression of heterologous genes, and its application for vaccination purposes has been suggested [15-17] Such an approach has also been developed for the YF 17D virus [18,19].

With regard to vaccine development, the insertion of larger gene fragments is indeed of interest, as it would allow the simultaneous expression of a number of epitopes. Given the difficulties in regenerating the YF 17D virus with longer genome insertions (more than 36 amino acids; prM-E replacements are not considered here as insertions), be it in between viral protease cleavage sites or in the 3' NTR, we have established a new method for the generation of live flaviviruses bearing whole gene insertions between the E and NS1 protein genes. Although conceptually similar to the methodology first proposed for insertions at viral protease cleavage sites [10], the cleavage between E and NS1 is carried out by the cellular signal peptidase present in the lumen of endoplasmic reticulum where virus maturation takes place. Therefore, a series of different structural elements are required to allow the recovery of infectious viruses with whole-gene insertions at this site.

The last 100 amino acids of the flavivirus E protein have been designated as the stem-anchor region [20] and are not part of the ectodomain for which the dimer structure has been established [21]. The stem region would electrostatically accommodate the inferior surface of the E ectodomain and the phospholipids of the external membrane layer [22]. It is made up of two helices and a connecting segment. The first helix (H1) forms an angle with the external membrane lipid layer whereas H2 rests on the outside with its hydrophobic side directed towards the hydrophobic membrane core [22,23].

The anchor region remains associated to the ER membrane through two antiparallel alpha helical transmembrane hydrophobic domains [TM1 and 2; [22]]. TM1 would serve as an anchor to E whereas TM2 would act as a signal sequence for NS1, and interactions between the two have a role in viral envelope formation [24]. The segment connecting TM1 and 2 has been shown to vary in amino acid sequence and length among the *Flaviviridae*, suggesting specific interactions [25]. Length and hydrophobicity of transmembrane domains as well as the charges of flanking amino acids and their structural arrangement may affect the topology of the secreted protein in the membrane [26]. Therefore, gene insertions between E and NS1 are likely to disrupt this functional arrangement if the design of such insertions does not contemplate the complex interactions among the different domains.

Herein we describe the design, construction and regeneration of live YF 17D and 17D-Dengue 4 (YF17D/DEN4) viruses bearing the green fluorescent protein gene between E and NS1. We have characterized foreign gene expression and genetic stability as well as recombinant virus immunogenicity.

## Results

### Design Of The Strategy For The Recovery Of Infectious Yf 17D Virus Bearing Genetic Insertions Between E And Ns1

**Figure 1:**
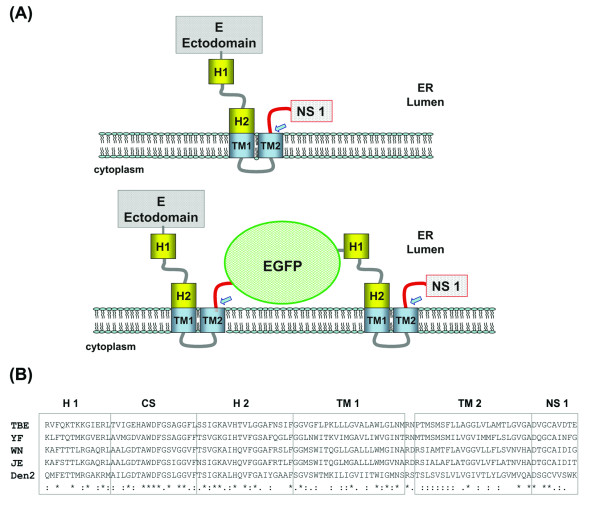
Topological arrangement of the flavivirus E stem-anchor region and its elements. The top panel (A) depicts the topology of part the polyprotein precursor (E-NS1) of YF virus, its insertion at the endoplasmic reticulum (ER) membrane, the expected proteolytic cleavage by the ER signal peptidase (blue arrow) and the flavivirus stem-anchor region with its different elements (H1 and H2; TM1 and TM2). The lower part of panel (A) illustrates the same region bearing the Enhanced Green Fluorescent Protein gene (EGFP). The EGFP protein is fused at its amino-terminus with nine amino acids of YF 17D NS1 protein and with the YF 17D E stem-anchor region at its carboxi-terminus. Blue arrows indicated ER signal peptidase cleavage sites Panel (B) presents the sequence alignment (Clustal W method) of the stem-anchor regions of flavivirus E proteins and the first nine amino acids of the NS1 protein amino-terminus (TBE; GenBank U27495; YF; GenBank U17066; JE; GenBank M18370; Den 2; GenBank M19197). Under the alignment, the following symbols denote the degree of conservation observed at each amino acid position: (*) identical in all sequences; (:) conserved substitutions and (.) semi-conserved substitutions.

For the flaviviruses, the polyprotein precursor transverses the ER membrane at various points being proteolytically processed in the ER lumen by cellular signal peptidases and in the cytoplasmic side by viral NS2B/NS3 protease. Protein secretion and processing require the presence of functional motifs. The design of a foreign sequence insertion in the YF 17D virus E and NS1 intergenic region considered the presence of such motifs as well as amino acid sequence conservation flanking this location. Figure 1A depicts the topology of the structural envelope protein E and the non-structural protein NS1. The E protein remains associated to the ER membrane through two anti-parallel alpha helical transmembrane hydrophobic domains (TM1 and 2; Fig. 1A).

Figure 1B displays a comparison of the amino acid sequences of the flavivirus E protein stem-anchor region and the amino-terminus of NS1 protein. This alignment was the basis for the identification at the amino acid level of the regions corresponding to each of the different segments in the stem (H1, CS e H2) and anchor (TM1 e TM2). Furthermore, the amino-terminus of NS1 also exhibited a strong conservation of 3 amino acids (Fig. 1B), which are likely to play a role in recognition, active site binding and proteolytic cleavage by the signal peptidase.

Our approach towards the regeneration of viable virus with a gene insertion between E and NS1 was to duplicate the first 9 amino acids of NS1 at the amino-terminus of the EGFP gene and the whole E protein stem-anchor domain at its carboxi-terminus (Fig. 1A). This structural arrangement of the EGFP expression cassette should allow the correct orientation for protein secretion towards the ER lumen, formation and folding in the ER of the E protein stem-anchor region as well as the appropriate orientation and cleavage at the amino-terminus of NS1. The insertion of EGFP gene in the chimeric YF17D/DEN4 genome followed the same strategy with the DEN4 E protein keeping its original stem-anchor region and the EGFP gene with the stem-anchor region of YF 17D virus.

### Recovery of YF17D/Esa/5.1glic recombinant virus and foreign gene expression

*In vitro *transcribed RNA was used to transfect cultured Vero cells. When the cytophatic effect (CPE) was widespread, the viability of the constructs could be visualized by fluorescence microscopy of the Vero cell monolayers. In the case of the YF17D/Esa/5.1glic virus it was performed at 72 h post-infection. This viral stock, called P1, was used for a second passage in Vero cells, or P2, which resulted in a viral stock with the titer of 6.18 log_10 _PFU/mL

### Growth and plaque morphology of YF 17D viruses

**Figure 2:**
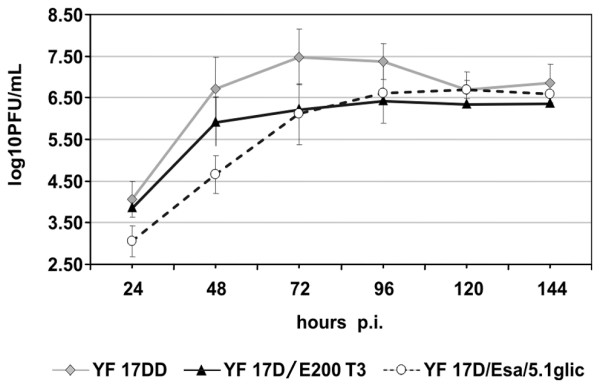
Viral growth curves in Vero cells. Cells were infected with either the control YF 17DD (gray lozenges) and YF17D/E200T3 (black triangles) viruses or the recombinant YF17D/Esa/5.1glic virus (open circles) at MOI of 0.02. Each time-point represents the average titer obtained from three separate experiments with the respective standard deviations.

The growth capacity of the recombinant YF17D/Esa/5.1glic virus was assessed comparatively to two other viruses, YF 17DD vaccine and YF17D/E200T3 [6]. Three independent experiments of virus growth in Vero cell monolayers were carried out and the results are shown in Figure 2. All experiments were carried out at low MOI according to requirements for viral vaccine production from certified seed lots.

At 24 h, 120 h and 144 h time points there were no significant difference between the viral titers of YF 17DD vaccine virus and YF17D/Esa/5.1glic (t-test; *P *= 0.095; *P *= 0.576 and *P *= 0.3890, respectively). But at 48 h, 72 h and 96 h the differences in virus yields were statistically significantly (*P *= 0.001; *P *= 0.004 and *P *= 0.043, respectively). The recombinant YF17D/Esa/5.1glic virus displayed a small plaque phenotype (0.99 ± 0.2 mm) when compared to the intermediate size of YF17D/E200T3 (1.65 ± 0.3 mm) and the large plaques of the YF 17DD virus (2.80 ± 0.7 mm).

### Expression of EGFP by recombinant YF 17D virus

**Figure 3:**
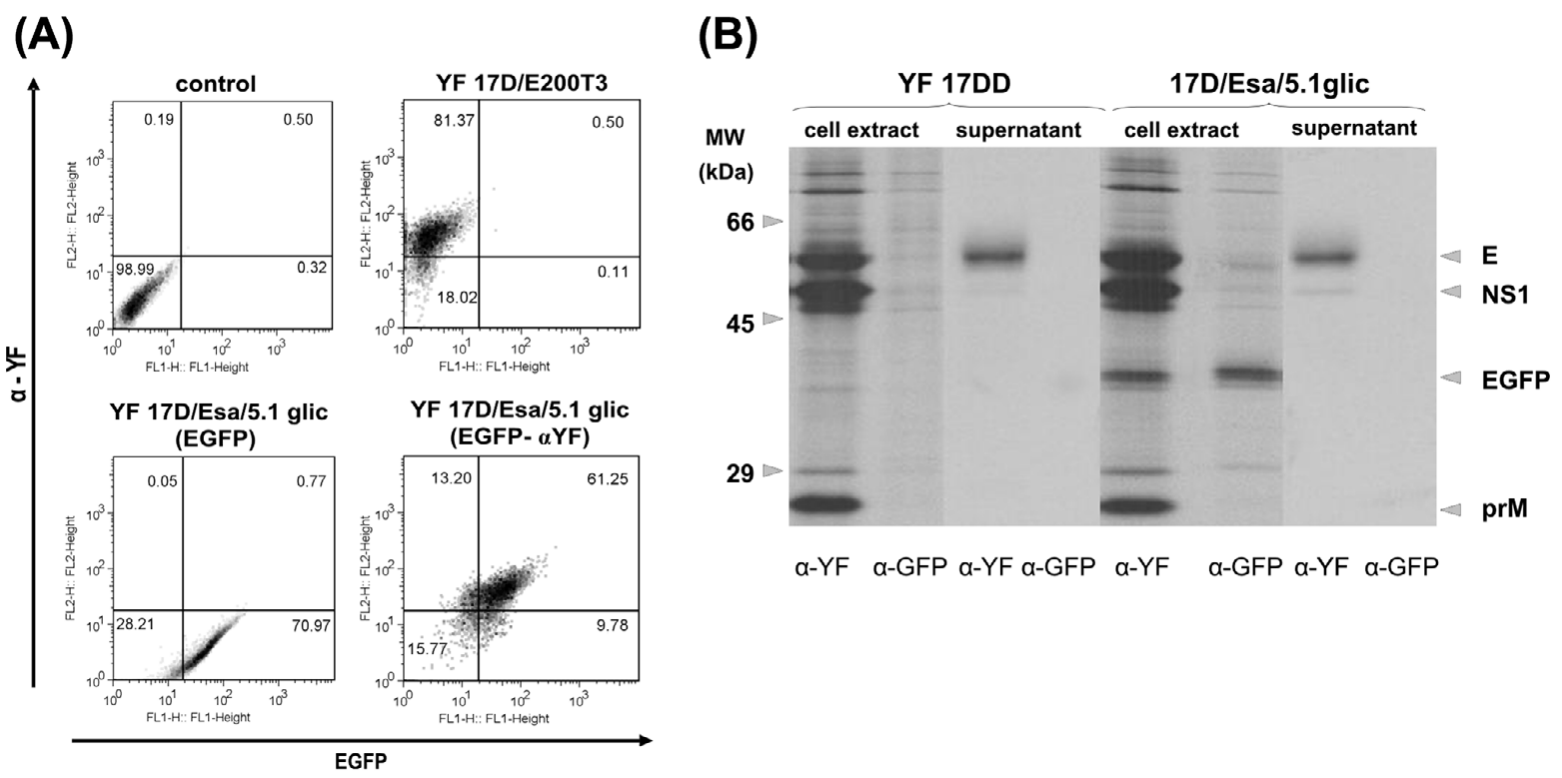
Analysis of the EGFP expression in YF 17D virus-infected Vero cells. (A) Flow citometry analysis at 72 h – post infection. Dot plots show the expression of YF antigens detected by intracellular staining with murine hyperimmune serum against YF virus (α-YF; *y*-axis) and of EGFP by direct detection of its fluorescence (EGFP; *x*-axis). The controls consisted of cells infected with no virus (control) and the parental virus (YF17D/E200T3). Cells infected by the recombinant virus were labeled (EGFP- α-YF) or (EGFP) only. The percentages of gated cell populations are indicated in each plot. (B) Immunoprecipitation profiles of protein extracts from supernatant and infected Vero cells with either YF 17DD or YF 17D/Esa/5.1glic viruses. These samples were immunoprecipitated with murine hyperimmune serum against yellow fever virus (α-YF) or rabbit polyclonal antiserum directed to EGFP (α-EGFP). Molecular weight markers are indicated on the left side of the figure whereas viral and recombinant proteins are identified on the right side.

We have approached EGFP expression in infected Vero cell monolayers by flow cytometry analysis (Fig. 3A). The EGFP expression together with viral antigens was highest between 72 and 96 hours post-infection. Figure 3A shows that EGFP expression was specific to Vero cells infected with the YF17D/Esa/5.1glic virus. At 96 h post-infection, 61 % of cells were expressing EGFP as well as viral antigens. These results indicated that the recombinant YF17D/Esa/5.1glic virus was capable of directing the expression of significant amounts of the heterologous protein even in cell cultures infected at low multiplicity (MOI of 0.02), pointing out the ability of the virus to disseminate to adjacent cells.

The expression of all viral proteins was monitored by immunoprecipitation (Fig. 3B). Radiolabelled lysates of virus-infected Vero cells were immunoprecipitated under non-denaturing conditions with EGFP or YF-specific sera and analyzed by SDS-PAGE. The immunoprecipitation patterns revealed that prM, E, NS1, NS3 and NS5 proteins of both recombinant YF17D/Esa/5.1glic and YF 17DD viruses co-migrated. An additional band corresponding to an apparent molecular weight (MW) of 35 kDa was observed in protein extracts from Vero cells infected solely with YF17D/Esa/5.1glic (Fig. 3B). This band corresponds to EGFP containing the stem-anchor region and was specifically recognized by an anti-GFP serum (Fig. 3B). This protein was also immunoprecipitated by the YF antiserum from YF17D/Esa/5.1glic-infected Vero cells (Fig. 3B). Since cell lysis and immunoprecipitation were carried out under non-denaturing conditions, membrane-bound viral proteins present in membrane- detergent micelles due to their amphyphatic character were recognized by YF polyclonal antiserum and immunoprecipitated. The EGFP, which is likely to be membrane-bound due to the stem-anchor region, could have been non-specifically carried along with other viral antigens during immunoprecipitation. Additionally, it was not possible to detect in both YF polyclonal antiserum and EGFP monoclonal antibody immunoprecipitation profiles higher molecular weight bands corresponding to non-proteolytic processed products, such as E-EGFP-NS1, E-EGFP and EGFP-NS1. It suggested the complete processing of the polyprotein precursor in this region. Moreover, pulse-chase experiments did not reveal the presence of such kind of non-processed proteins (data not shown). The analysis of the infected cell culture supernatant revealed only E protein and traces of NS1, suggesting that EGFP was retained inside the cell.

**Figure 4:**
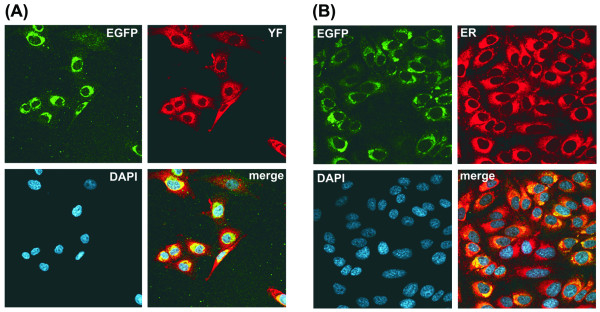
Intracellular localization of the recombinant EGFP protein. (A) Co-localization of viral antigens and EGFP. Infected cells were fixed with 4% paraformaldehyde, permeabilized with 0.5% Triton X-100, and processed for immunolabeling. The designation on the upper right corner indicates the localization of the heterologous protein (EGFP); (α-YF) corresponds to the same cells stained with a hyperimmune antiserum to YF virus proteins; (DAPI) represents DAPI-stained cell nuclei; (merge) co-localization assessed by spectral overlap (yellow in right down panel) of the images of this preparation. (B) Co-localization of EGFP and the ER compartment. Live infected cells were labeled with ER-Tracker Red (Molecular Probes) and fixed in 4% paraformaldehyde. (EGFP) localization of heterologous protein; (ER) cells labeled with ER marker; (DAPI) nuclei counterstained with DAPI; (merge) co-localization assessed by spectral overlap (yellow in right down panels) of the images of this preparation.

To determine the intracellular location of the EGFP protein expressed by the YF17D/Esa/5.1glic virus we initially performed an indirect fluorescence assay in infected Vero cell monolayers, which were fixed, permeabilized and stained with a polyclonal antiserum against YF viral antigens (Fig. 4A). The staining of YF antigens spread from the perinuclear region to a reticular network through the cytoplasm whereas EGFP was located in the perinuclear area (Fig. 4A). The intracellular location of EGFP could be better observed by co-localization with an ER marker, ER-Tracker Red, in infected Vero cells (Fig. 4B). It was possible to confirm that the EGFP subcellular location overlapped with the ER labeled area and that this protein accumulated in the perinuclear region of the ER (Fig. 4B). This set of results strongly indicate that the heterologous protein (EGFP) expressed by the recombinant YF virus is not secreted from the infected cells and is mainly associated with the ER compartment.

### Immunogenicity for mice of YF 17D viruses

We have next asked the question whether the recombinant virus was able to elicit an immunological response against the YF virus and the foreign protein. For this purpose groups of 4-week old BALB/c mice were immunized subcutaneously with two doses of approximately 5.0 log_10 _PFU of each virus. Fifteen days after the last dose mice were bled and neutralizing antibodies to YF measured by PRNT.

Table 1 shows that both the YF 17D vaccine virus and the YF17D/Esa/5.1glic recombinant virus were capable of eliciting significant titers of neutralizing antibodies to YF. All animals seroconverted to YF virus after subcutaneous inoculation with either virus. For YF17D/Esa/5.1glic virus the antibody titers ranged from 1:37 to 1:211 (GMT of 1:80) whereas those elicited by the YF 17DD vaccine virus varied from 1:45 to 1: 308 (GMT of 1:140). The titers of neutralizing antibodies to the YF 17DD virus in immunized animals were significantly higher than those found for the group of animals inoculated with YF17D/Esa/5.1glic virus (t test; *P *< 0.02). It is noteworthy that the immunization with YF 17D/Esa/5.1glic virus elicited antibodies against EGFP in 80 % of the animals with titers varying from 26 to 3,535 ng/mL (GMT of 158 ng/mL; Table 1).

**Table 1:** Immunogenicity of YF17D/Esa/5.1glic for BALB/c mice.

Immunogen	Animals (*n*)	PRNT_50_*			ELISA-EGFP***		
		
		% Sero-conversion	GMT ± SD	Titer Range**	% Sero-conversion	GMT ± SD	Titer Range
YF 17DD	15	100	140 ± 80	45 – 308	0	< 16	< 16
YF17D/Esa/5.1glic	20	100	80 ± 47	37 – 211	80	158 ± 1,144	26 – 3,535
199 Earle's Medium	15	0	< 10	< 10	0	< 16	< 16

* Reciprocal of the dilution yielding 50% plaque reduction.

** Differences in the titers of neutralizing antibodies virus in animals immunized with YF 17DD and YF17D/Esa/5.1glic were statistically significant (t test; *P *< 0.02).

***The titer of antibodies directed against EGFP was calculated based on standard curves of a monoclonal antibody specific to GFP and is expressed in ng/mL.

### Genetic stability of the YF 17D/Esa/5.1glic virus

**Figure 5:**
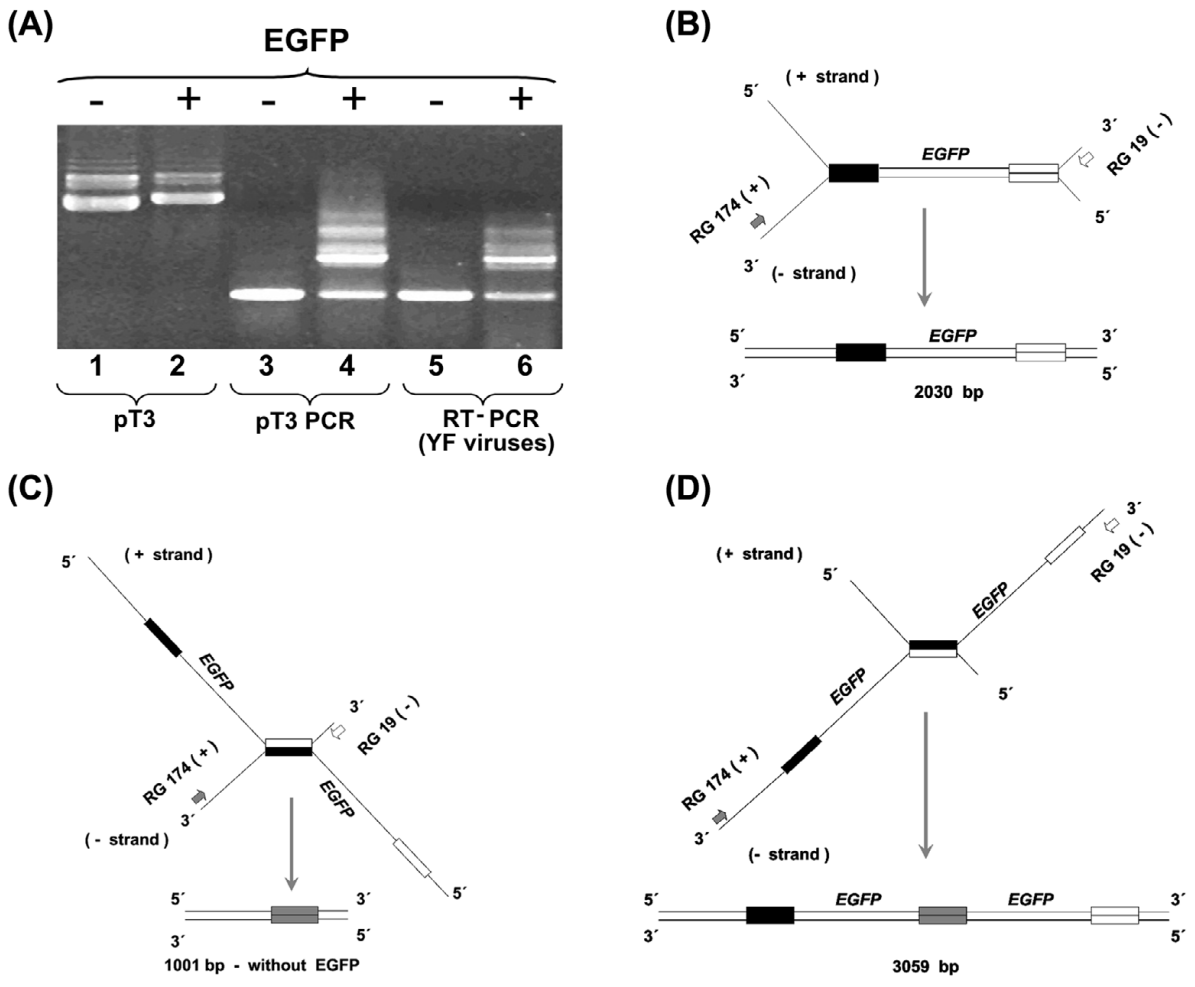
Viral genetic stability and artifactual DNA amplification of the EGFP gene. (A) Agarose gel electrophoresis of plasmid T3 DNA without and with the EGFP cassete (lanes 1 and 2, respectively); DNA amplification of plasmid T3 and the recombinant one (lanes 3 and 4, respectively); RT-PCR on RNA of YF17D/E200T3 and YF17D/Esa/5.1glic 2P viruses without and with the EGFP cassete (lanes 5 and 6, respectively). (B) Schematic representation of the amplification based on the correct annealing of the E protein gene (black bars) and the EGFP stem-anchor (white bars) domains from two different DNA strands yielding an amplicon of 2,030 bp. (C) and (D) schematic representation of the amplification based on the spurious alternative annealing possibilities of the E protein gene (black bars) and the EGFP stem-anchor (white bars) regions from two different DNA strands yielding amplicons of 1,001 bp (without the EGFP cassete and with a single stem-anchor domain, gray bars) or 3,059 bp (with the duplicated EGFP gene and an extra copy of stem-anchor region), respectively.

Genetic insertions between the E and NS1 genes of recombinant YF 17D viruses must be stable if this strategy is to be useful for the construction of new live attenuated vaccine viruses expressing antigens of other pathogens. We have initially evaluated the genetic stability of the YF17D/Esa/5.1glic virus insertion by RT-PCR amplification of the E-NS1 region of 2P virus (Fig. 5A). A DNA amplicon of 2,030 bp in length indicated that the cassete region was complete whereas smaller amplicons would be suggestive of genetic instability. Passage 2 (2P) displayed a diverse electrophoretic profile of amplicons, varying from 3.0 kb to 1.0 kb (Fig. 5A). This complex profile was also observed after amplification of a homogenous plasmid DNA preparation (based on its uniform migration in agarose gel and nucleotide sequence analysis), suggesting the complexity was not necessarily due to genomic rearrangements upon virus regeneration and an additional passage in Vero cells (Fig. 5A, lanes 1–4). The presence of the 1.0 kb amplicon, which is suggestive of EGFP gene deletion, and other amplicons longer than 2.0 kb were noted in all RT-PCR reactions using RNA from YF17D/Esa/5.1glic 2P virus or T3 Esa EGFP plasmid DNA (Fig. 5A). These are a consequence of spurious amplification during the bidirectional synthesis of the PCR reaction due to the presence of a direct repeat region of 315 nucleotides flanking the EGFP gene, which corresponds to the YF 17D virus E protein stem-anchor and NS1 N-terminal region duplication. So, the band corresponding to the correct recombinant genomic structure contains 2,030 bp and its amplification is explained by the pairing represented in Figure 5B. Alternatively, during the PCR reaction, the stem and anchor gene region of the heterologous EGFP cassete might hybridize with the homologous and non-allelic region, located at the complementary negative strand, corresponding to the E protein stem-anchor region (Fig. 5C). The resulting product would be shorter, with 1,001 bp in length, as it would not include the insertion cassete, and therefore, be equivalent to the vector virus E-NS1 gene region. On the other hand, the opposite situation could also occur, in which a 288-nucleotide alignment may occur in the region encoding the stem and anchor domain of the virus E protein with the negative strand complementary to the heterologous expression cassete. Accordingly, a longer PCR fragment (3,059 bp) would be produced including a duplicated EGFP gene (Fig. 5D), which in its turn, is also detected (Fig. 5A) after amplification of plasmid DNA and viral RNA, although with a lower intensity due to its less efficient synthesis. These interpretations are supported by the single 1,001 bp amplicon profile observed for plasmid and virus that do not contain the expression cassete, i.e., that have a single stem-anchor sequence. Therefore, the use of RT-PCR for genetic stability studies constituted only an initial evaluation to determine the maintenance of the heterologous EGFP cassette in the virus population.

**Figure 6:**
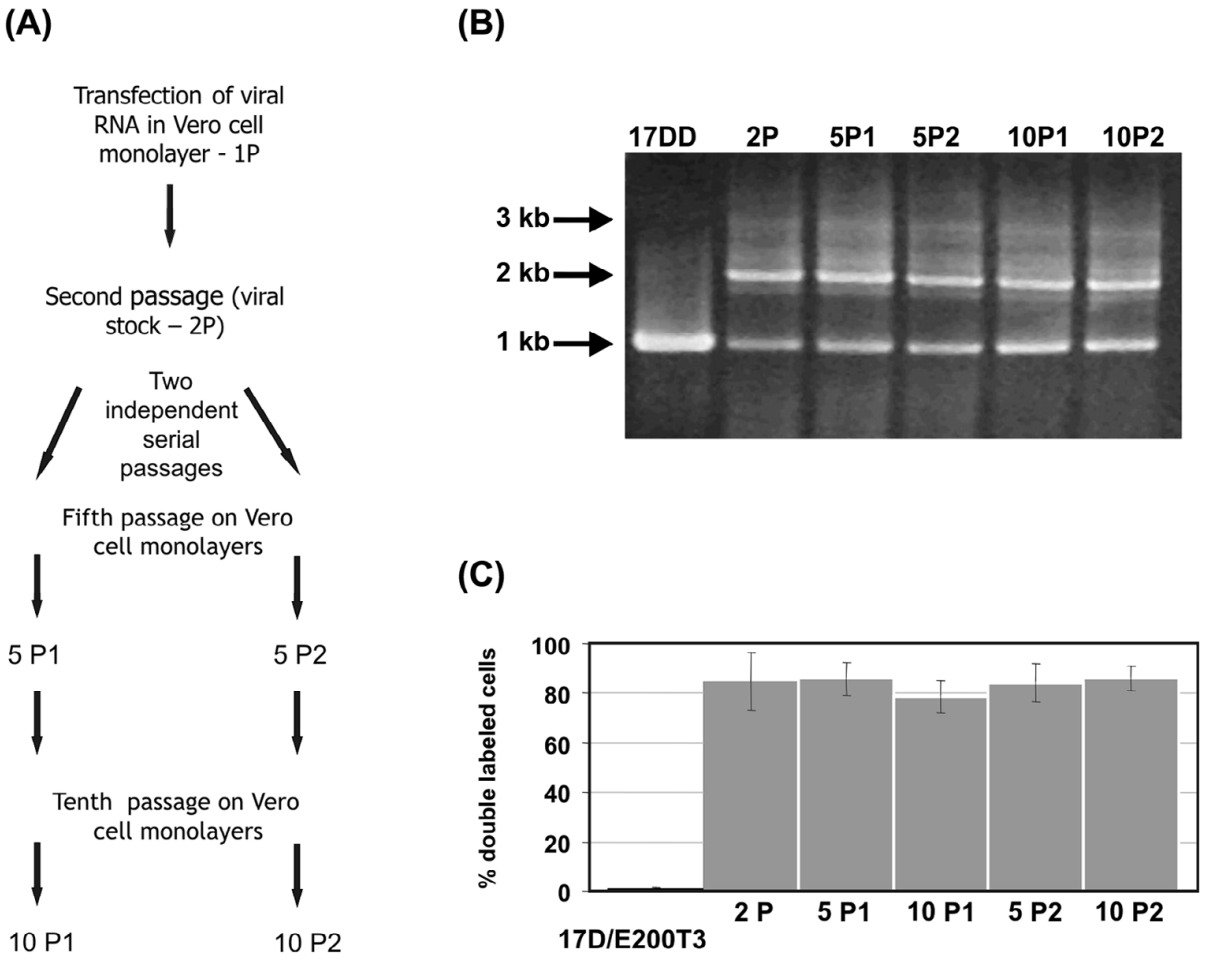
Analysis of recombinant virus genetic stability after serial passaging. (A) Schematics of viral regeneration and subsequent passages (10) of the YF 17D/Esa/5.1 glic virus obtained after RNA transfection. Two independent series of serial passages (at MOI of 0.02); P1 and P2 were analyzed by RT-PCR and flow citometry at passages 5 and 10 and are represented in all panels as 5P1, 10P1, 5P2 and 10P2. In these experiments the YF17D/E200-T3 virus was used as negative control for EGFP expression. (B) Electrophoretic analysis of RT-PCR amplicons from viral RNA extracted of samples from the supernatant of cultures used to derive the citometry data (C) according the passage history (A). The length of the main RT-PCR bands are shown on the left side. (C) The rate of double gated cells (YF+, EGFP+) over the total YF+ gated cells (YF+, EGFP+ plus YF+, EGFP- gated cells) corresponds to the percentage of cells infected by YF 17D/Esa/5.1 glic virus stably expressing the EGFP protein. The respective columns indicate the values for each of the viral passages.

We have studied the genetic stability of YF17D/Esa 5.1glic virus by two independent serial passages of this virus in Vero cells up to the tenth passage (Fig. 6A). We used infection low MOI. as to maximize the number of viral RNA replication cycles and thereby increase the chances for mutational events to take place. The cassete integrity in the viral genome was checked by RT-PCR analysis on RNA extracted from viral samples at different passage levels. Although the 2.0 kb amplicon, which corresponds to the complete heterologous expression cassete, was detected as far as the tenth consecutive passage (Fig. 6B) a smaller amplicon of 1.0 kb was also evident. In order to clarify whether distinct passage populations were composed of a mixture of viruses either carrying the entire heterologous cassete or deletions thereof, Vero cells infected with these viruses at different passage levels were submitted to flow cytometry analysis. Only 0.8% of the cells infected with the control virus YF17D/E200T3 showed double fluorescence (Fig. 6C), whereas 78 % to 86 % of cells after YF17D/Esa/5.1glic virus infection was positive for YF viral antigens and EGFP. This variation in the percentage of positive cells along the passages was not statistically significant (One-way ANOVA; *P *= 0.74). These results suggest the continuous presence of the EGFP gene in the recombinant virus genome and its expression throughout the passages. However, as we continued with these two independent serial passage lines in Vero cells up to the fifteenth one, it was possible to demonstrate a change in the total EGFP+ YF+ labeled cells, which varied from 83 % to 84 % at the tenth passage to 1 % and 20 %, at the fifteenth, respectively (data not shown).

To better characterize the genetic stability of the YF17D/Esa/5.1glic virus, we set up a serial passage experiment in Vero cells with 5 plaque purified viral clones. All Vero cell cultures infected with each of the 5 cloned viruses exhibited double EGFP and viral antigen fluorescence. The double fluorescence ratio varied from 95 to 99% in cells infected with cloned viruses at their fifth passage. But, at the tenth passage, two cloned viruses have exhibited a double labeling percentage of 7 % and 33 %, suggesting the continuous loss of the foreign sequence in this interval (data not shown). However, the other three cloned virus samples displayed 77 %, 93 % and 80 % of double gated cells at the tenth passage (data not shown), indicating again genetic stability of the EGFP-bearing recombinant virus population.

### Expression of EGFP by a chimeric flavivirus

**Figure 7:**
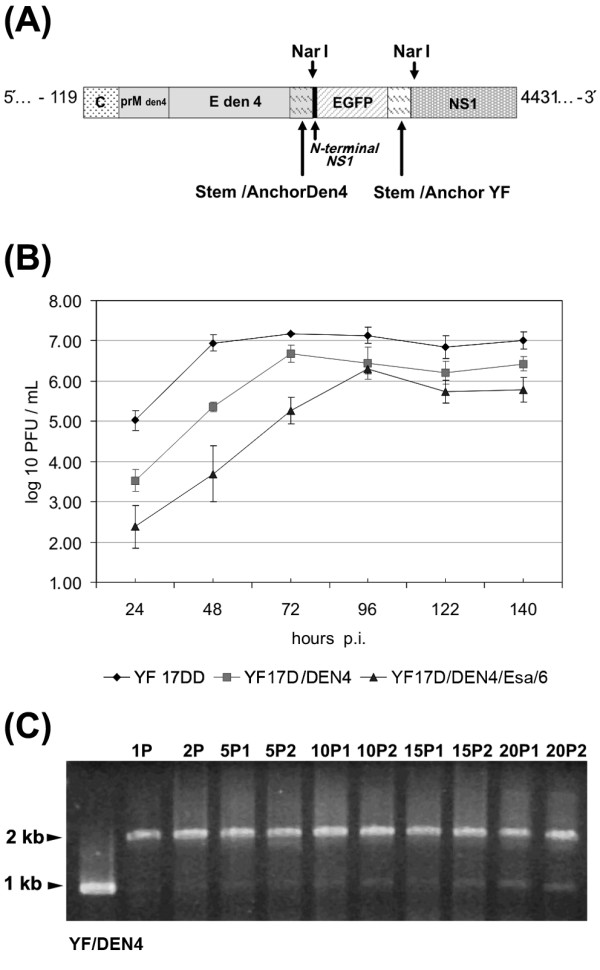
Molecular cloning of EGFP protein expression cassete in the chimeric YF17D/DEN4 virus genome. (A) Schematic representation of YF 17D/DEN4/Esa/EGFP/6 recombinant virus genome and the genetic elements fused to EGFP gene. (B) Growth of recombinant YF17D/DEN4 viruses in Vero cells. Three independent experiments were performed to measure viral spread in Vero cells after infection with an multiplicity of infection (MOI) of 0.02. Cell culture supernatant aliquots were taken at 24, 48, 72, 96, 120 and 140 hour post-infection (p.i.) and titrated by plaque formation on Vero cell monolayers. (C) Analysis of recombinant YF 17D/DEN4/Esa/6 virus genetic stability after serial passaging on Vero cell monolayers. Electrophoretic analysis of RT-PCR amplicons from viral RNA extracted from samples of the supernatant of cultures according to the passage numbering indicated on top of each lane. The first lane corresponds to cDNA-derived YF17D/DEN4 virus RNA; the remaining lanes are RT-PCR profiles from YF17D/DEN4/Esa/6 virus RNA at different passage levels with lanes 2 and 3 corresponding to amplicons from RNAs of viral stocks (1P, transfection supernatant) or passage two (2P, first passage of transfection supernatant), respectively. Lanes 4 to 11 represent RT-PCR products, which were obtained from viral RNA in the fifth, tenth, 15^th ^and 20^th ^passages of the two independent passage lineages (5P1 and 5P2; 10P1 and 10P2, 15P1 and 15P2, 20P1 and 20P2, respectively).

To verify whether this strategy might be applicable to clone foreign sequences in other flavivirus genomes, we have constructed a recombinant YF17D/DEN4/Esa/EGFP virus, in which the YF prM/E genes were replaced by the homologous genes of the DEN type 4 virus with the EGFP cassete being inserted in the same E/NS1 intergenic region (Fig. 7A). It is noteworthy that there were two stem-anchor regions: the first one located just upstream of the EGFP gene, corresponding to the stem anchor of the dengue 4 E protein gene, and the second one located just downstream of the EGFP gene, corresponding to the stem-anchor of the YF 17D virus E protein, as part of the heterologous expression cassete (Fig.7A). Viable YF 17D/DEN4/Esa/EGFP virus, designated YF17D/DEN4/Esa/6, was recovered after *in vitro *transcription and transfection of Vero cells with RNA. The chimeric YF17D/DEN 4/Esa/6 construct could only be recovered after trypsinization of the RNA-transfected cell monolayer with an additional incubation of 96 h when CPE became evident. This viral stock, called P1, was used for a second passage in Vero cells, or P2, with a titer of 6.48 log10 PFU/mL. Passage 2 virus was used for further analysis.

Aiming at the characterization of the growth capability of the YF/DEN4/Esa/6 virus in comparison to the YF 17DD vaccine virus and parental chimeric YF17D/DEN4 virus Vero cell monolayers were infected with these viruses at MOI of 0.02. The YF 17DD and 17D/DEN4 viruses peaked at 72 hours after infection, with titers of 7.2 ± 0.3 and 6.7 ± 0.4 log_10 _PFU/mL, respectively, while the recombinant YF17D/DEN4/Esa/6 virus, at 96 hours after infection displayed a viral titer of 6.3 ± 0.1 log_10 _PFU/mL (Figure 7B). At all the time points of the growth kinetic the titers of the recombinant EGFP YF/DEN4 virus were significantly different from the corresponding titers of the YF 17D vaccine virus (*t test; P *< 0.05).

The genetic stability of the chimeric YF17D/DEN4/Esa/6 virus was assessed by two series of independent passages in Vero cells up to the twentieth passage. The expected length of DNA amplicon containing the EGFP expression cassete is 2,046 bp, while the same region in the parental YF17D/DEN4 virus is 1,017 bp long. As can be observed in Figure 7C, the band that contains the heterologous insertion is maintained as far as the twentieth passage in both series, indicating viral genetic stability.

## Discussion

The yellow fever virus has been considered as an appealing viral vector for the development of new human vaccines [27]. The most successful approach so far has been the exchange of the YF viral envelope genes with those from other flaviviruses [9]. These chimeric viruses have been shown to be safe, and immunogenic and are undergoing clinical trials [28]. It would be desirable, however, the design of strategies for the insertion of foreign sequences and not only the replacement. In this regard short sequences encoding known B and T cell epitopes, have been inserted in the intergenic region between NS2B-NS3 and at a selected site of the E gene [6,8,10,11]. Although these YF recombinant viruses were immunogenic, attenuated and grew to high titers, foreign insertions longer than 40 codons were not genetically stable. As the E-NS1 region represents a functional shift in flavivirus genome from the structural to non-structural genes, insertions of larger gene fragments at this intergenic site might induce fewer disturbances in the virus cycle as compared to other sites.

During viral RNA translation, the flavivirus polyprotein precursor transverses the ER membrane at various points being proteolytically processed in the ER lumen by cellular signalases and at the cytoplasmic side by the viral NS2B/NS3 protease [29]. The E protein remains associated to the ER membrane through two transmembrane domains (TM1 and TM2). TM2 would also act as a signal sequence for NS1 secretion. The stem region that connects the E protein ectodomain to the transmembrane domains consists of the two helices accommodating the inferior surface of the E ectodomain and the external membrane layer [22]. With regard to the signalase cleavage sites, the carboxi-terminus of flavivirus E protein is composed of the VXA motif frequently present in other eukaryotic processing sites [30]. The DXGC amino acid sequence present at the amino-terminal of NS1 is much conserved among flavivirus. The temporal and spatial coordination of the viral precursor protein processing is critical for virus morphogenesis [31], and probably also to the assembly of the viral replication complex. Thus, our design of the expression cassette in which the E protein stem-anchor and a short segment of NS1 were added to the heterologous sequence contemplates polyprotein processing and secretion into the ER, processes that are fundamental to viral viability. Notwithstanding a reduced growth rate as compared to the original YF 17D vaccine virus, the recombinant YF 17D/Esa/5.1glic and YF17D/DEN4/Esa/6 virus yields are still suitable for industrial vaccine production.

Recombinant YF 17D viruses bearing genetic insertions between the E and NS1 genes must be stable to be useful for the development of new live attenuated vaccine viruses expressing antigens of other pathogens. The genetic stability of the EGFP expression cassette was studied in YF17D/Esa/5.1glic and YF17D/DEN4/Esa/6 viral samples submitted to serial cell passages. Cells were infected at low MOI (0.02) as this would force high replication rates for the viral genome thereby allowing recombination events to take place possibly leading to cassette removal. Nevertheless, these viruses were genetically stable as far as maintenance of the heterologous cassette is concerned up to the tenth continuous cultivation (YF17D/Esa/5.1glic) and the 20^th ^passage (YF17D/DEN4/Esa/6). The flow cytometry data for cells infected with YF17D/Esa/5.1glic supports the genetic stability of the insert up to the tenth passage. It is possible to produce seed lots intended for industrial production starting from cDNA with 4 passages [32].

The apparent instability revealed by PCR analyses of the viral E-NS1 genomic region might be related to the presence of a 288 nt direct repeat flanking the foreign gene, which corresponds to the duplicated E protein stem-anchor region. It is known that the flavivirus RNA is synthesized semi conservatively and uses double-stranded RNA as replicative form [33]. Thus, it is conceivable that pairing between the stem-anchor complementary non-allelic 288-nucleotide sequences might lead to viral RNA copies with EGFP gene deletions, with these mutants prevailing in viral populations after some rounds of cell passaging. Consistent with this hypothesis is the observation that the YF17D/DEN4/Esa/6 is genetically more stable. This is probably due to the presence of two divergent stem-anchor domains with reduced nucleotide pairing during RNA replication.

Recombinant YF 17D viruses bearing prM-E from other flaviviruses have been suggested as new vaccines [9]. In areas with extensive vaccination to YF these chimeric viruses expressing foreign antigens from the E-NS1 site would be useful to overcome immunity to YF. We have shown here the viability of one such chimera bearing the EGFP gene. Interestingly the YF17D/DEN4/Esa/6 virus was more stable than the YF17D/Esa 5.1glic virus, as the EGFP insertion in this chimeric virus could be detected up to the twentieth serial passage on Vero cell monolayers. The fact that the genome of this virus contains two divergent stem-anchor regions, one from DEN 4 virus E gene and the other from YF 17D virus E gene, which share only 58 % of nucleotide sequence homology suggests that the lower the homology of the stem-anchor regions, the higher the stability of the recombinant viral genome. A complete assessment of the genetic stability of a new YF 17D recombinant virus bearing the EGFP gene fused to the DEN4 stem-anchor sequence is underway using serial passaging followed by antigen expression monitoring and viral RNA amplification. This analysis should highlight the true stability of insertions between E and NS1 to confirm that for this strategy to render genetically stable viruses it is important to use the stem anchor domains of different flaviviruses.

The foreign EGFP expressed by the recombinant YF 17D virus remained cell associated, since it was not possible to detect it in infected cell culture supernatant, but only in cell extracts. The same methodology allowed the successful detection of YF NS1 secretion in different cell types [34]. Moreover, EGFP was located within ER compartment as shown by confocal microscopy. The presence of the YF 17D E protein stem-anchor region at its carboxi-terminus is likely to have allowed its anchoring in the luminal side of the ER membrane. It has been shown that intracellular prM and E are mostly localized to the ER as stable heterodimers [35] and heterodimer formation is likely to depend on the accumulation of these proteins in the ER. Interestingly specific ER retention signals have been suggested to exist in the TM1 domain [36]. The association of stem-anchor region with the reporter gene EGFP provides an experimental system to study flavivirus protein trafficking within the infected cell. The insertion of marker genes into the flavivirus genome may also allow *in vivo *studies on viral cell and tissue tropism as well as cellular processes related to infection.

Flaviviruses are assembled in the ER membranes and virions released by exiting through the Golgi compartment. This process involves hypertrophy of the ER membranes, due to virus particle accumulation [37] and contributes to ER stress [38] and apoptosis induction. YF 17D and wild type viruses can induce apoptosis in immature dendritic cells and hepatocytes [11,39]. The phagocytosis of apoptotic cells infected with YF recombinant virus by macrophages might have allowed EGFP peptide presentation through HLA class II molecules eliciting T cell CD4+ responses. This type of response would favor an IgG antibody response to the foreign protein, and this is exactly the type of molecules detected in the ELISA test. On the other hand, infected cell necrosis might result in local inflammation, leading to the B-cell activation. Both alternatives would explain why mouse immunization with the recombinant YF17D/Esa/EGFP 5.1 glic virus elicited antibodies to EGFP even with the foreign protein retained in the cell ER. Studies on the duration of the antibody response and immunoglobulin isotyping may highlight the predominant mechanism.

One of the hallmarks of YF 17D vaccine is its extremely low incidence of adverse events. All YF 17D viruses retain a certain degree of neurovirulence for mice and monkeys. We have shown that the neurovirulence of the YF17D/Esa/EGFP and YF 17D/DEN4/Esa/6 recombinant viruses was not exacerbated for mice further warranting the potential of this approach to new live virus vaccine development (data not shown). Final proof for the attenuation of YF 17D recombinant viruses bearing insertions at the E-NS1 region will have to come from the monkey neurovirulence test, which constitutes the ultimate standard established to ensure the attenuation of any YF 17D virus intended for human use [40].

It is noteworthy that a recombinant YF 17D virus expressing a precursor of the Lassa virus glycoprotein between YF 17D E and NS1 genes has been recently described [41]. This construct differs from our design since only the 23 carboxi-terminal hydrophobic amino acids corresponding to the TM2 domain of the YFV 17D E gene were duplicated downstream of the LASV GPC gene to serve as a signal sequence to ensure insertion of the YFV 17D NS1 protein into the ER. However, the proteolytic processing of the Lassa virus protein precursor was not appropriate due to the lack of an amino terminal hydrophobic domain. Moreover, no evidence for YF and foreign antigen trafficking in the infected cell was presented. This recombinant replicated poorly in guinea pigs but still elicited antibodies against both viruses as measured by Elisa tests. Deficient immune responses, as a consequence of non optimal genome structure and polyprotein processing and trafficking ending with low levels of antigen may explain the partial protection observed in the challenge experiments [41]. It was claimed that YF 17D-Lassa recombinant virus growth was comparable to that of the parental 17D vaccine virus but no data was shown and there was no experimental evidence for its genetic stability.

The flavivirus genome is small and compact. Any modification may have a deleterious effect in RNA replication, polyprotein precursor processing or viral protein function, with unpredictable burden on viral capability to replicate in the vertebrate animal host and therefore to elicit the robust immune response characteristic of YF 17D virus [1]. In this regard the work described by Bredenbeek et al and herein is rather complementary towards the definition of the best strategy to engineering the 17D virus to express larger foreign protein domains. However, our strategy is likely to be useful for a broader live attenuated YF 17D virus-based vaccine development for other diseases since recombinant viruses expressing protozoan and other viral antigens of interest have been developed.

## Conclusion

We were able to express a reporter autofluorescent protein in the intergenic E/NS1 region of YF 17D virus. The methodology is based on the duplication and fusion of the functional motifs flanking the E and NS1 intergenic region to the exogenous gene. It allowed the correct processing of the viral polyprotein precursor and did not compromise substantially the viral viability. The heterologous cassette was genetically stable up to the tenth continuous cultivation in the case of YF 17D virus and to the 20th passage the twentieth passage for the YF17D/DEN4 virus, suggesting that the lower homology of the stem anchor region the higher the genetic stability of the recombinant virus.

The foreign EGFP expressed by the recombinant YF 17D virus remained cell associated and could be localized to the RE compartment. The YF recombinant virus was capable of eliciting significant titers of neutralizing antibodies to YF and also antibodies against EGFP.

This system is likely to be useful for a broader live attenuated YF 17D virus-based vaccine development for human diseases. Moreover, insertion of foreign genes into the flavivirus genome may also allow *in vivo *studies on flavivirus cell and tissue tropism as well as cellular processes related to flavivirus infection

## Materials and methods

### Cell cultures

Vero cells, originally obtained from ATCC, were grown in Earle's199 medium supplemented with 5% fetal calf serum (FCS).

### Construction of infectious cDNA clones

The generation of chimeric E/NS1 regions with the EGFP gene was done by PCR-PCR amplification. The first fragment (783 base pairs; bp) was amplified with positive primer RG328 (5'CTAGGAGTTGGCGCCGATCAAGGATGCGCCATCAACTTTGGCGTGAGCAAGGGCGAGGAGCT 3') that contained the last 15 nucleotides of E plus the initial 27 of the NS1 gene (positions 2,453 to 2,479; based on Gene Bank accession number X03700) and 20 nucleotides from EGFP. The negative stranded oligonucleotide RG329 (5'GCCTTTCATGGTCT GAGTGAACAACTTCTTGTACAGCTCGTCCATGCCGAG 3') contained the last 24 nucleotides of the EGFP gene plus the initial 15 nucleotides corresponding to the amino-terminal domain of the E protein stem-anchor region. This amplification was carried out on plasmid pEGFP-C2 (Clontech) with *Pfx *DNA Polymerase according to the manufacturer (Invitrogen).

The second fragment (339 bp) was based on the amplification of the YF T3 plasmid [6] with oligonucleotides RG330 (5'CTCGGCATGG ACGAGCTGTACAAGAAGTTGTTCACTCAGACCATGAAAGGC 3') and RG331 (5'GCCAAAGTTGATGGCGCATCCTTGATCGGCGCCAACTCCTAGAGAC 3'). This fragment included 24 nucleotides from the carboxi-terminal of the EGFP gene followed by the YF or DEN4 stem-anchor region (288 bp; YF nucleotides 2,165 to 2,452; Gene Bank accession number U17066) and 27 nucleotides from the amino-terminus of the NS1 gene (as above).

Both fragments were mixed in equimolar amounts and reamplified with 20 μM of RG328 and RG331 oligonucleotides. All amplifications were obtained with Platinum *Pfx *DNA Polymerase (Invitrogen) according to manufacturer specifications. The resulting fragment of 1,071 bp was purified with silica-based kit (Qiagen) and cloned in the pGEM-T plasmid (Promega) using chemically competent *E. coli *MC1061. The insert was removed by digestion with Nar I, purified from agarose gel as above and ligated into YF T3 plasmid using T4 DNA ligase (Invitrogen). This ligation was used to transform chemically competent *E. coli *Sure cells (Stratagene). Recombinant plasmids were screened by digestion with NarI to confirm the insertion and its orientation was verified by nucleotide sequencing. This led to the identification of pT3 Esa EGFP. This plasmid contains the middle part of the YF genome and served later to reconstitute the full genome by ligation with the extreme 5' and 3' ends derived from plasmid E200 [6]. Both plasmids, pE200 and pT3, corresponded to the parental genetic background of the recombinant YF virus construct and were employed to generate a parental control virus called YF17D/E200T3. This virus differs from YF 17D at nucleotides 1568, 1570, 8526 and 8808 [6]. The chimeric virus YF17D/DEN4 has the prM/E genes of dengue 4 virus, and its construction will be described elsewhere. The full-length chimeric genome was cloned in pACNR1180 plasmid bearing or not the EGFP gene between E and NS1 genes [6].

### Recovery of virus from cloned cDNA: transcription and transfection

We have prepared two templates by *in vitro *ligation [42] of DNA fragments from pE200, pT3 and pT3Esa EGFP plasmids [6]. For the template with the pT3Esa EGFP plasmid we utilized a version of pE200 bearing a N-linked glycosylation motif at position E154 of the envelope protein. These templates (E200T3 and E200glic T3 Esa EGFP together with a full-length YF17D/DEN4-EGFP plasmid) were digested with XhoI, transcribed by SP6 RNA polymerase (AmpliScribe SP6, Epicentre Technologies) and RNA preparations transfected into Vero cells with LipofectAmine (Invitrogen) as previously described [43]. The recovered viruses were designated YF17D/E200T3, YF17D/Esa/5.1glic and YF/DEN4/Esa/6, respectively. Viral stocks (P2) were prepared by infecting Vero cell monolayers with the virus present in the supernatant resulting from transfection (P1) with a multiplicity of infection (MOI) of 0.1. The P2 viruses were used for all characterizations.

Viral growth and plaque size characterization. Viral growth curves were determined by infecting monolayers of Vero cells at MOI of 0.02. Cells were seeded at a density of 62,500 cell/cm2 and infected 24 h later. Samples of cell culture supernatant were collected at 24-hour intervals post-infection. Viral yields were estimated by plaque titration on Vero cells. Plaque size was determined by growing viruses in Vero cells seeded at 62,500 cells/cm2 in six-well plates with an overlay of 3 mL 0.5% low melting point agarose (Promega) in 199 medium supplemented with 5% fetal bovine serum. Following 4 days of incubation at 37°C, 2 ml of medium supplemented with 0.1% neutral red was added and the plates were incubated for one more day prior to fixation. Two YF 17D viruses with different plaquing properties were used as controls: YF17D/E200 T3 with an intermediate plaque and the YF17D/14 virus with a large plaque phenotype [6]. Two experiments were carried out and the values were derived from counting 20 plaques for each virus in each assay. The different time points of the growth curves were compared using t test (GraphPad Prism 3.02 Program). The differences were considered significant when *P *< 0.05.

### Flow cytometry

Cell samples were obtained from infected monolayers (at MOI. of 0.02) by trypsin treatment, centrifugation (350 g, 5 min) and washing with PBS pH 7.4 supplemented with 1 % BSA and 0.01% sodium azide (PBS-BSA-NaN_3_). Afterwards, Vero cells were adjusted to 10^6 ^cells/tube. Cells were fixed in PBS-BSA-NaN_3 _with 1% paraformaldehyde for 20 min at 4°C and further washed twice in PBS, before permeabilization for 20 min at 4°C with PBS-BSA- NaN_3 _containing 0.15 % saponin (Sigma Chemical Co). Cells were washed once with PBS-BSA- NaN_3 _and incubated with yellow fever (17D) polyclonal hyperimmune mouse ascetic fluid (NIAID) diluted to 1:100 in PBS-BSA- NaN_3_-saponin for 60 min at 4°C. Cells were washed again and treated with polyclonal goat anti-mouse immunoglobulins labeled with R-phycoerytrin (PE; DakoCytomation) for 30 min at 4°C. Stained cells, were washed in PBS-BSA- NaN_3_-saponin, resuspended in PBS-BSA- NaN_3 _with 1% paraformaldehyde and kept at 4°C up to three days until acquisition (10,000 events) in a FACScalibur flow cytometer (BD Biosciences). Data was analyzed using FlowJo 7.2 Software (TreeStar Inc.). Genetic stability analysis was derived from FACS data as the percentage of double positive (EGFP+ or α-YF +) gated cells over the total α-YF + antigen cells (EGFP+ and α-YF + gated cells plus α-YF + gated cells). Data was collected from three independent experiments. One-way ANOVA was performed to compare the experimental groups using GraphPad Prism (version 3.00 for Windows, GraphPad Software, San Diego California USA). The differences were considered significant when *P *< 0.05.

### RT/PCR and sequencing

Cell culture supernatants were utilized for viral RNA extraction with Trizol LS (Invitrogen) and RNA precipitated with isopropanol in the presence of glycogen (Invitrogen). Amplification of the viral genomic E-NS1 region encompassing the heterologous insert was performed essentially as described previously [44]. The RNA was used as template for cDNA synthesis with a negative strand YF-specific synthetic oligonucleotide (genome position 2619 – 2639), followed by PCR amplification with the GeneAmp 9600 instrument (Applied Biosystems) and the GeneAmp RNA PCR Core Kit (Applied Biosystems) with the addition of a positive YF-specific primer (genome position 1639 – 1659). Amplification products were further purified from excess primers with silica-based kits (QIAGEN). These products were sequenced directly without molecular cloning. Nucleotide sequencing reactions were performed with the BigDye terminator mix version 3.1 (Applied Biosystems) according to manufacturer's recommendations. Electrophoresis of fluorescent products was performed in an ABI PRISM 3730 instrument (Applied Biosystems). Nucleotide sequences were analyzed using Chromas software version 2.3 (Technelysium Pty Ltd) and a consensus sequence for each viral genome was derived from contiguous sequences with SeqMan II software from Lasergene package version 5.07 (DNAStar Inc.).

### Genetic stability assay

Recombinant viruses were submitted to two independent series of ten passages each in Vero cells at MOI of 0.02. In the fifth and tenth passages, the Vero cell monolayers at 72 h post-infection were recovered for flow cytometry analysis to determine EGFP and YF antigen expression. Viral RNA was extracted from the culture supernatants with Trizol LS, cDNA synthesized and sequenced as described above.

### Confocal immunofluorescence microscopy

Vero cells grown on 8-well Lab-Tek Chamber Slides (Nunc) at a density of 20,000 cells/cm^2 ^were infected at a MOI of 0.1 with Earle's199 medium alone (mock infected), or with control virus YF17D/E200T3 and the recombinant virus YF17D/Esa/5.1glic. Seventy-two hours post-infection, the cell monolayers were fixed with 4% paraformaldehyde- phosphate buffer 0.1 M pH 7.8 for 10 min at room temperature. The cells were permeabilized with PBS containing 0.5% Triton X-100 for 10 min and further incubated in blocking buffer (PBS containing 3% BSA) for 30 min at room temperature. Both primary- (YF 17D polyclonal hyperimmune mouse ascitic fluid-NIAID; diluted 1:80 and secondary antibody (Alexia Fluor 546 goat anti-mouse IgG -Invitrogen; diluted 1:400) incubations were carried out for 30 min at room temperature. Alternatively, cells were *in vivo *labeled in Hank's Balanced Salt Solution with calcium and magnesium (HBSS/Ca/Mg, GIBCO) containing 800 nM ER-Tracker Red (Molecular Probes) for 30 min at 37°C. The cells were fixed as described above and washed three times with HBSS buffer. Both preparations were treated with SlowFadeGold antifade reagent with DAPI (Invitrogen). Confocal microscopy was performed with a Carl Zeiss confocal laser-scanning microscope, model LSM 510 META and the image capture was achieved with the help of the LSM Image Browser 3.5 Software.

### Metabolic labelling and immunoprecipitation

Vero cells were infected at a multiplicity of 0.5 PFU/cell in 60 mm dishes (cell density of 62,500 cells/cm^2^). After a 72 hour incubation, the cells were starved in methionine-free Dulbecco's Modified Eagle Medium (DMEM) for 20 min and labeled with 30 μCi Redivue [^35^S]methionine (GE Healthcare Life Sciences) for one hour. The cells were washed once and incubated with 10 mL Earle's 199 medium with 5% FCS for 3 h at 37°C in a CO_2 _incubator. The supernatants were removed, centrifuged 5 min at 300 g at 4°C and protease inhibitors were added (PMSF 0.1 mM; leupeptin 10 μM; aprotinin 25 μg/ml). The adherent cells were scraped off and lysed under nondenaturing conditions in the presence of the same protease inhibitors.

The volume of 250 μL of cell extracts and 1 mL of the cell culture supernatant of each sample were immunoprecipitated with mouse polyclonal hyperimmune ascitic fluid to YF 17D (NIAID) and a rabbit polyclonal antiserum directed against GFP (Clontech). Immunoprecipitates were fractionated with protein A-agarose (Invitrogen) and analyzed by 10% SDS-PAGE. Gels were treated with sodium salicylate for fluorographic detection.

### Immunogenicity of YF 17D viruses in mice

Groups of ten four-week old BALB/c mice (CEMIB, UNICAMP) were subcutaneously injected with two doses of 100,000 PFU in 100 μL of YF17D/Esa/5.1glic or YF 17DD viruses with an interval of 15 days. Two weeks after the last immunization, mice were bled from the retrorbital vein, serum samples were treated for 30 minutes at 56°C and stored at -20°C. YF neutralizing antibody titer was determined by plaque reduction neutralization test (PRNT_50_) [45]. The values of neutralizing antibody titers of each experimental group were compared using t test (GraphPad Prism 3.02 Program). The differences were considered significant when *P *< 0.05.

Antibodies to the EGFP heterologous protein were detected by ELISA using microtiter plates (Costar) coated with 10 ng/well of the recombinant GFP of *Aequoria victoria *(Clontech) diluted in 100 μL carbonate buffer 0.05 M pH 9.6. After overnight incubation at room temperature, the plates were washed three times with phosphate-buffered saline containing 0.05% (v/v) of Tween-20 (PBS-Tween), and blocked at 37°C for two hours with PBS containing 5% (w/v) non-fat milk with 1% (w/v) of bovine serum albumin (BSA, Sigma Co.). The assays were performed in duplicate and every plate was composed of the respective pooled sera of each experimental group. The positive and negative controls consisted respectively of the mouse monoclonal IgG2a antibody (JL-8) specific for GFP and pooled sera from animals immunized with culture medium (Clontech). Pooled sera were analyzed in serial dilutions from 1:20 to 1:2,560, and the JL-8 monoclonal antibody was employed in the range from 10 to 0.78 ng. After a two-hour incubation at room temperature, unbound antibodies were washed away with PBS-Tween, and diluted 1:500 peroxidase-conjugated goat anti-mouse IgG heavy and light chain (Kirkegaard and Perry), was added to each well. After one-hour incubation at room temperature, the excess labeled antibody was removed by washing, and the reaction was developed with o-phenylenediamine (Sigma Co.) and 1 μL/ml H_2_O_2 _(Merck). After 15 min, 2 M H_2_SO_4 _solution was added to stop the reaction and the plates were read at 492 nm on VERSAmax ELISA reader (Molecular Devices). Every ELISA plate contained a positive column of serially diluted JL-8 monoclonal antibody, which provided the standard curve. The different standard curves were analyzed by linear regression to check the linearity of the data and then used to determine the titers in the experimental groups. Therefore, the EGFP antibody titers were expressed in ng/mL based upon the curve established for the JL-8 monoclonal antibody specific to EGFP.

All animal studies were carried out according to a protocol reviewed and approved by the Institutional Committee for Experimentation and Care of Research Animals (CEUA-FIOCRUZ: P0112/02).

## Competing interests

The author(s) have declared that the present methodology is the subject of a patent application having as authors MCB and RG and the Oswaldo Cruz Foundation as the sponsoring institution.

## Authors' contributions

MCB designed the viral constructions and the study, coordinated the study and drafted the manuscript; SMM carried out the YF virus genome cloning work and the flow cytometry analysis; GFT performed the confocal microscopy analysis and the EGFP-ELISA studies; AAR was engaged in the YF/DEN4 virus construction and related studies; ASD was responsible for nucleotide sequencing, assisted in animal studies and helped with the ELISA analysis; PJO performed neutralization plaque assays and data analysis; MSF discussed and assisted in animal studies; CFK designed flow cytometry analysis and led data interpretation; RG designed the viral constructions and helped to coordinate the study and manuscript draft. All authors read and approved the final manuscript.
